# Complementary Dynamics of Banana Root Colonization by the Plant Growth-Promoting Rhizobacteria *Bacillus amyloliquefaciens* Bs006 and *Pseudomonas palleroniana* Ps006 at Spatial and Temporal Scales

**DOI:** 10.1007/s00248-020-01571-0

**Published:** 2020-08-11

**Authors:** Rocío Margarita Gamez, Sandra Ramirez, Martha Montes, Massimiliano Cardinale

**Affiliations:** 1 Corporación Colombiana de Investigación Agropecuaria – Agrosavia, C.I. Turipaná, Montería, Cordoba Colombia; 2 Corporación Colombiana de Investigación Agropecuaria – Agrosavia, C.I. Tibaitatá, Mosquera, Cundinamarca Colombia; 3 Corporación Colombiana de Investigación Agropecuaria – Agrosavia, C.I. Caribia, Zona Bananera, Magdalena Colombia; 4 Institute of Applied Microbiology, Justus-Liebig-University Giessen, Giessen, Germany; 5 Department of Biological and Environmental Sciences and Technologies – DiSTeBA, University of Salento, Centro Ecotekne - via Provinciale Lecce-Monteroni, I-73100 Lecce, Italy

**Keywords:** Banana cv. Williams, Plant growth-promoting rhizobacteria (PGPR), *Bacillus*, *Pseudomonas*, Fluorescence in situ hybridization-confocal laser scanning microscopy (FISH-CLSM), Colonization dynamics

## Abstract

Banana (*Musa acuminata*) growth for commercial purposes requires high amounts of chemical fertilizers, generating high costs and deleterious effects on the environment. In a previous study, we demonstrated that two plant growth-promoting rhizobacteria (PGPR), *Bacillus amyloliquefaciens* Bs006 and *Pseudomonas palleroniana* Ps006, isolated in Colombia, could partially replace chemical fertilizers for banana seedling growth. In a second work, the effects of the two inoculants on banana transcripts were found to occur at different times, earlier for Bs006 and later for Ps006. This leads to the hypothesis that the two rhizobacteria have different colonization dynamics. Accordingly, the aim of this work was to analyze the dynamics of root colonization of the two PGPR, Bs006 and Ps006, on banana growth over a time frame of 30 days. We used fluorescence in situ hybridization (FISH) and confocal laser scanning microscopy (CLSM), followed by three-dimensional reconstruction and quantitative image analysis. *Bacillus amyloliquefaciens* Bs006 abundantly colonized banana roots earlier (from 1 to 48 h), ectophytically on the rhizoplane, and then decreased. *Pseudomonas palleroniana* Ps006 was initially scarce, but after 96 h it increased dramatically and became clearly endophytic. Here we identify and discuss the potential genetic factors responsible for this complementary behavior. This information is crucial for optimizing the formulation of an effective biofertilizer for banana and its inoculation strategy.

**Electronic supplementary material:**

The online version of this article (10.1007/s00248-020-01571-0) contains supplementary material, which is available to authorized users.

## Introduction

Approximately 5.6 million hectares of land are dedicated to banana production globally, according to the latest Food and Agriculture Organization Corporate Statistical Database (FAOSTAT) available data from 2017 [1]. Large amounts of fertilizers are required to maintain high yields [2], representing high costs and a potential environmental threat. Biofertilizers are environmentally-friendly alternatives to chemical fertilization for the stimulation of plant growth. They are based on beneficial rhizobacteria, usually designated as plant growth-promoting rhizobacteria (PGPR). These microbial species applied to seeds, seedlings, or leaves can increase seed germination, plant biomass, and crop yield [2–10]. Treatments with PGPR have been used to reduce germination time and increase the growth of various crops including vegetables [11–13] and fruits such as apricot, cherry, raspberry, apple, and banana [14–19].

The plant growth-promoting mechanisms of rhizobacteria are based on either direct interaction with the host plant or indirectly via antagonistic activity against phytopathogens [20, 21]. Direct mechanisms include the ability to produce plant hormones (auxins [22, 23], cytokinins [24], gibberellins [25], and ethylene [24]), symbiotic and asymbiotic N_2_ fixation [8, 26], inorganic phosphate solubilization, and mineralization of organic phosphates and other nutrients, or both [25, 27]. Indirect mechanisms (antagonism against phytopathogens) can be achieved by siderophore production, synthesis of antibiotics or cell wall-degrading enzymes, competition for binding sites, and the recently discovered root-hair endophyte stacking (RHESt) mechanism [28–31].

Among the effective PGPR, strains of *Bacillus* and *Pseudomonas* have been widely studied in various crops, and some commercial biofertilizers are based on species belonging to both genera. For several years, we have been conducting research to identify suitable PGPR to be used as beneficial inoculants on banana cv. Williams, which is of great economic interest for several banana-producing countries, including Colombia. In a previous study [32], we selected the strains *Bacillus amyloliquefaciens* Bs006 and *Pseudomonas palleroniana* Ps006 (formerly *Pseudomonas fluorescens* Ps006) as the best-performing PGPR among a group of potential candidates isolated in Colombia. In another study [33], we investigated the effect of these two strains on banana by plant transcriptomics. We observed that the effects occurred at different moments, being Bs006 stronger at an earlier stage, while Ps006 induced more changes later. *B. amyloliquefaciens* Bs006 and *P. palleroniana* Ps006 stimulated differential gene expression involved in plant growth promotion at different times, indicating that they use different metabolic pathways. These observations lead to the hypothesis that the two strains might colonize banana roots with different dynamics and different behaviors at both temporal and spatial scales.

In the current study, we tested this hypothesis as follows. We investigated the dynamics of banana root colonization by these two strains over a time frame of 30 days after inoculation, expecting the following: (i) *B. amyloliquefaciens* Bs006 and *P. palleroniana* Ps006 colonize banana roots with different dynamics over the considered time scale (Bs006 earlier, Ps006 later), and (ii) both PGPR colonize different microniches of banana roots at a spatial scale. Fluorescence in situ hybridization (FISH) staining [34] coupled with confocal laser scanning microscopy (FISH-CLSM) was used as a detection method, as it enables accurate localization of bacteria in the root habitat, as well as a reliable characterization of the colonization patterns and dynamics [35]. Finally, we performed an in silico genome analysis of the strains Bs006 and Ps006 to compare their genetic background in terms of genes involved in plant growth-promoting activity and plant–microbe interactions, to identify the genetic basis potentially responsible for the different colonization patterns.

## Materials and Methods

### Plant Material

Banana plants (*Musa acuminata* Colla) of the cultivar “Williams” (Cavendish subgroup, genome AAA) were used in this study. The mother plants were obtained from the ex vitro Musa Bank of the Caribia Research Center of the Corporación Colombiana de Investigación Agropecuaria (AGROSAVIA), formerly known as CORPOICA, Colombia. Murashige and Skoog (MS) culture medium [36], supplemented with 0.1 g l^−1^ myo-inositol, 3 ppm (ppm) benzyl amino purine (BAP), 0.5 ppm indole acetic acid (IAA), 1 ppm thiamine hydrochloride, and 30 g l^−1^ sucrose at pH 5.7, was used for seedling micropropagation in the laboratory by meristem extraction and in vitro propagation/rooting of the seedlings. An in-house AGROSAVIA standardized protocol developed to provide local small farmers with high-quality plants was used. From a healthy banana plant, a basal corm (weight of 300–400 g) was extracted; successive cuts, washes, and disinfection with detergent powder, iodine, and chlorine were carried out until only the apical meristem (~1 cm^2^) remained for micropropagation (Fig. S1). After leaf emergence (usually after 15 days), the seedlings were transferred to a growth chamber for an additional 4 weeks of growth under the following conditions: 23/20 °C ± 1 °C day/night temperature, 16/8-h light/dark photoperiod, and 65% ± 10% relative humidity (Fig. S1).

### Bacterial Inoculants

Two rhizobacteria strains, *B. amyloliquefaciens* Bs006 and *P. palleroniana* Ps006, obtained from the AGROSAVIA microbial collection were used. These strains, isolated in Colombia from *Physalis peruviana* and *Furcraea andina*, respectively, were already identified at the genome level [37, 38] and were selected as the best-performing plant growth promoters on banana in a previous study [32]. They are part of the AGROSAVIA bank of microorganisms. Initially [38], strain Ps006 had been classified as *Pseudomonas fluorescens*; however, in July 2019 it was reclassified by the NCBI (www.ncbi.nlm.nih.gov/nuccore/LRMR00000000). The strain Bs006 is currently undergoing a reclassification into the species *Bacillus velezensis* [39]. Nonetheless, the process is still under consideration, and therefore in this manuscript we kept it as *B. amyloliquefaciens*.

### Planting, Microbial Inoculation, and Sampling

Seedlings 1–3 cm in height with three fully developed leaves and adequate root development were selected for transplantation into a new culture medium. After in vitro rooting, the seedlings were inoculated with 5 ml of the microbial inoculants *B. amyloliquefaciens* Bs006 and *P. palleroniana* Ps006. Control plants (without microbial amendment) were inoculated with 5 ml of sterile distilled water. Three treatments were evaluated: (1) plants inoculated with *B. amyloliquefaciens* Bs006, (2) plants inoculated with *P. palleroniana* Ps006, and (3) control plants without inoculum. For each treatment, three biological replicates, corresponding to plants grown in different containers, were analyzed. Five sampling times were chosen: 1 h, 48 h, 96 h, 15 days, and 30 days, assigned codes T1, T2, T3, T4, and T5, respectively. Samples were fixed for FISH analysis immediately after sampling.

### Fluorescence In Situ Hybridization and Confocal Laser Scanning Microscopy (FISH-CLSM) Analysis

FISH staining was performed following the “in-tube FISH” protocol [40]. The bacterial EUB338MIX universal probe [41] labeled with Cy3 was used to stain the bacterial cells on inoculated and uninoculated roots. As a FISH negative control, subsamples of inoculated roots were hybridized with a Cy3-labeled NONEUB probe [42].

Stained samples were preliminary checked under a Zeiss Axioplan fluorescence microscope (Zeiss, Jena, Germany), using the Filter Set 15. They were then extensively observed with the Leica SP8 confocal laser system (Leica Microsystems, Wetzlar, Germany). The 561 nm laser light was used for the excitation of Cy3, and emitted light was detected in the range of 565–620 nm; the root tissue was excited with 405 nm laser light, and the emitted autofluorescence was detected in the range of 428–510 nm. The two signals were combined in multicolored images, where the inoculated bacteria were observed in red and the root tissue in cyan. For each bacterium and time point, three root samples were comprehensively observed. Three representative confocal series were then acquired using the 63× objective, with a step of 0.45–0.80 μm between the confocal planes, and analyzed using Imaris 8.1 software (Bitplane, Zürich, Switzerland). Each confocal series contained between 4 and 65 optical slices (two channel images per optical slice at a resolution of 1024*1024 dpi), for a total of 1602 individual images analyzed. Three-dimensional models were created by converting bacterial signals into spheres and root tissues into isosurfaces. The number of spheres and root volumes were measured automatically by Imaris software and used to calculate the cell density as the number of cells/cm^3^ of root tissue. The cell densities of the two strains over the whole time frame were compared by Spearman’s rho correlation (the variable did not show a normal distribution), using SPSS version 20 software (IBM Corporation, Armonk, NY, USA). The Adobe Photoshop CS6 program (Adobe Systems Inc., San Jose, CA, USA) was used to assemble and label the final images.

### In Silico Analysis of the Genome for Plant Growth-Promoting Activity

#### Bacterial DNA Extraction and Amplification

The SureSelect^QXT^ Target Enrichment system for the Illumina multiplexed sequencing kit (Agilent Technologies, Santa Clara, CA, USA) was used to obtain the genome sequence. A volume of 2 μl of DNA (concentration of 25 ng μl^−1^) was mixed with the kit buffer and the Herculase enzyme to generate enzymatic fragmentation; the adapters were then added to the ends of the fragments in a single reaction. The DNA libraries were amplified by polymerase chain reacion (PCR) using labeled primers; the amplified libraries were purified with AMPure XP pearls (Beckman Coulter, Brea, CA, USA). Finally, the quantity and quality of the DNA were tested with the Agilent 2100 Bioanalyzer (Agilent Technologies, Inc.), using a high-sensitivity DNA assay. Fragment length ranged between 300 and 2000 bp.

#### Sanger Sequencing of the Complete Bacterial Genomes

The PCR product was purified by mixing 5 μl of the amplified product with 2 μl of ExoSAP-IT™ PCR Product Cleanup Reagent (Applied Biosystems, Foster City, CA, USA), and incubating at 37 °C for 60 min and at 80 °C for 15 min. Sequencing was performed with the BigDye™ Terminator v3.1 Cycle Sequencing Kit (Applied Biosystems), by mixing 4 μl of the kit mixture, 2 μl of each of the forward or reverse primers (3.2 μM) included in the kit, 2 μl of the purified PCR product, and 2 μl of molecular-grade water. The PCR program began with denaturation at 96 °C for 1 min, followed by 25 cycles (10 s at 96 °C, 5 s at 50 °C, and 4 min at 60 °C). The sequencing product was purified with the BigDye XTerminator™ Purification Kit (Applied Biosystems), taking 10 μl of the XTerminator solution and mixing with 12 μl of the sequencing product and 45 μl S-adenosylmethionine (SAM) buffer. The mixture was stirred in a vortex mixer for 30 min and then centrifuged at 12,000×*g* for 2 min. A volume of 10–12 μl of the purified sequencing product was extracted and run on an ABI PRISM 3100 analyzer (Applied Biosystems).

#### Characterization of Bacterial Genomes

Full genome sequencing was performed using the HiScan™SQ system (Illumina, San Diego, CA, USA), generating 10,785,126 individual readings of 150 bp in length. The genomes of *B. amyloliquefaciens* Bs006 and *P. palleroniana* PS006 were assembled using ARGO, a reference-guided assembler developed at NCBI, and SPAdes, a de novo assembler [43]. The draft genome was annotated using the Rapid Annotation using Subsystem Technology (RAST) server and the nr database, whilst antimicrobial resistance genes (ARGs) were identified using the SARG 2.0 database, RAST server, and nr database. Genome drafts were previously announced in Gamez et al. in 2015 and 2016 [37, 38]. The COGs (Clusters of Orthologous Groups of proteins) categories were identified using the eggNOG-mapper tool (http://eggnog-mapper.embl.de/) employing the default options [44].

To quantify the number of genes/factors occurring in the genomes of the two strains involved in functions that are relevant in plant–microbe interactions, the online tool PIFAR (Plant–bacteria Interaction Factors Resource) was used [45].

Secondary metabolites biosynthetic clusters were detected with the antiSMASH tool (https://antismash.secondarymetabolites.org), using the default setting, and “strict” detection strengthness [46].

## Results

### FISH-CLSM Analysis of Colonized Roots

**Fig. 1:**
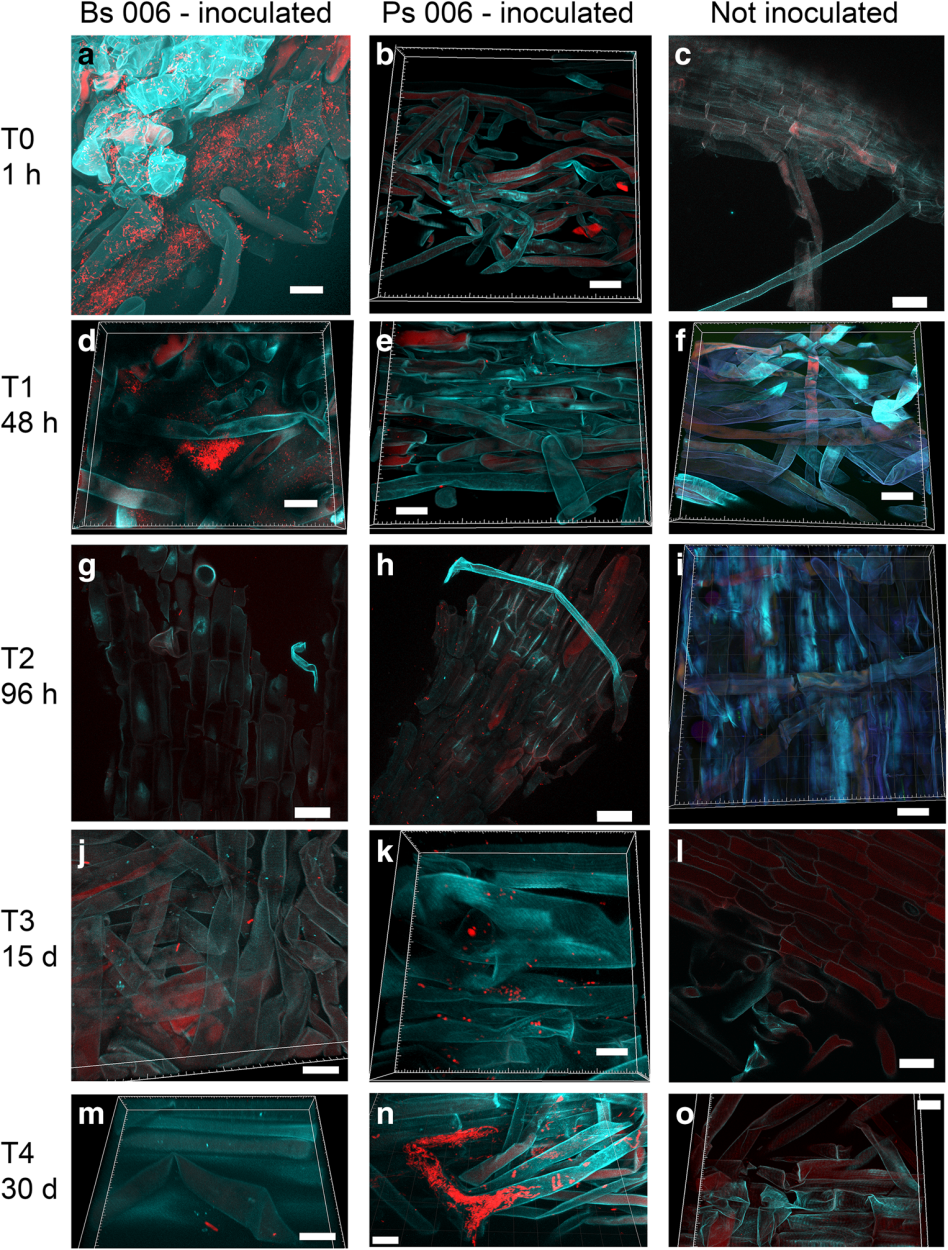
Confocal microscopy series of FISH-stained samples showing the colonization dynamics of *Bacillus amyloliquefaciens* Bs006 and *Pseudomonas palleroniana* Ps006 on banana roots at different time points. Volume-rendered images were created with Imaris software. Images of non-inoculated roots without detectable bacterial signals are also shown. Scale bars: **a**, **d**, **f**, **i**, **j**, **k**, **n**, **o** = 20 μm; **b**, **c**, **e**, **g**, **h**, **l** = 30 μm; **m** = 10 μm

**Fig. 2:**
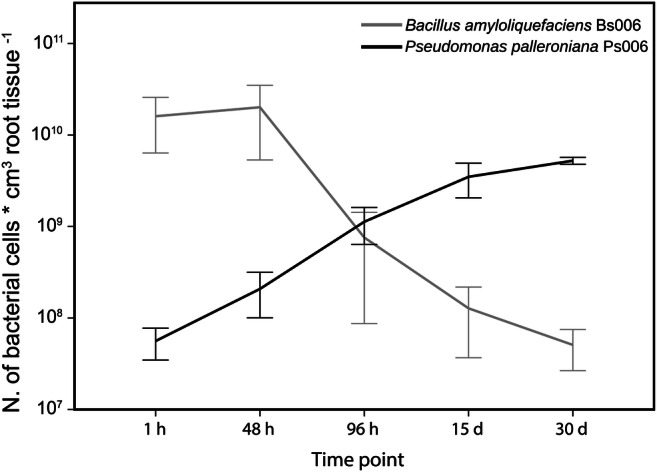
Quantification of the bacterial cell density in the banana root obtained by quantitative analysis of the confocal series after conversion of the original FISH-conferred signals into spheres (bacteria) and isosurfaces (root tissue)

**Fig. 3:**
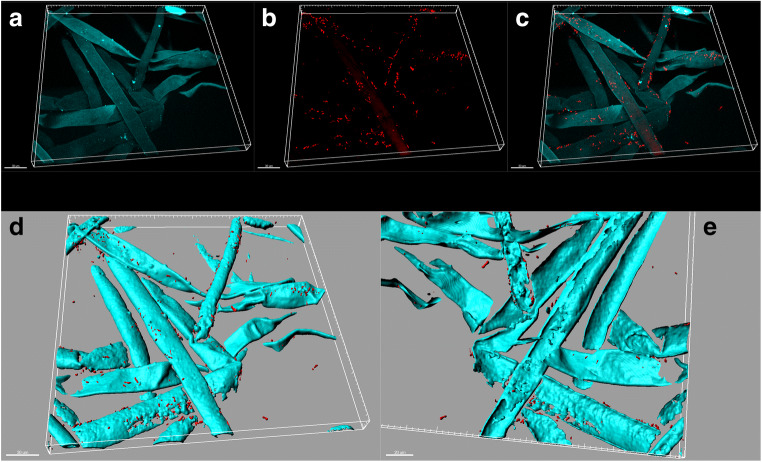
FISH-CLSM images showing the epiphytic colonization of banana root hairs by *Pseudomonas palleroniana* Ps006 1 h after inoculum. **a** Root tissue autofluorescence (cyan); **b** signal of the Cy3-labeled EUB338MIX universal probe for bacteria (red); **c** overlay of **a**–**b**; **d** three-dimensional model of **c**, showing bacteria as spheres and root tissue as isosurfaces; **e** flipped view of **d** showing no bacterial cells inside the root hairs. Scale bars: **a**–**d** 30 μm; **e** 20 μm

**Fig. 4:**
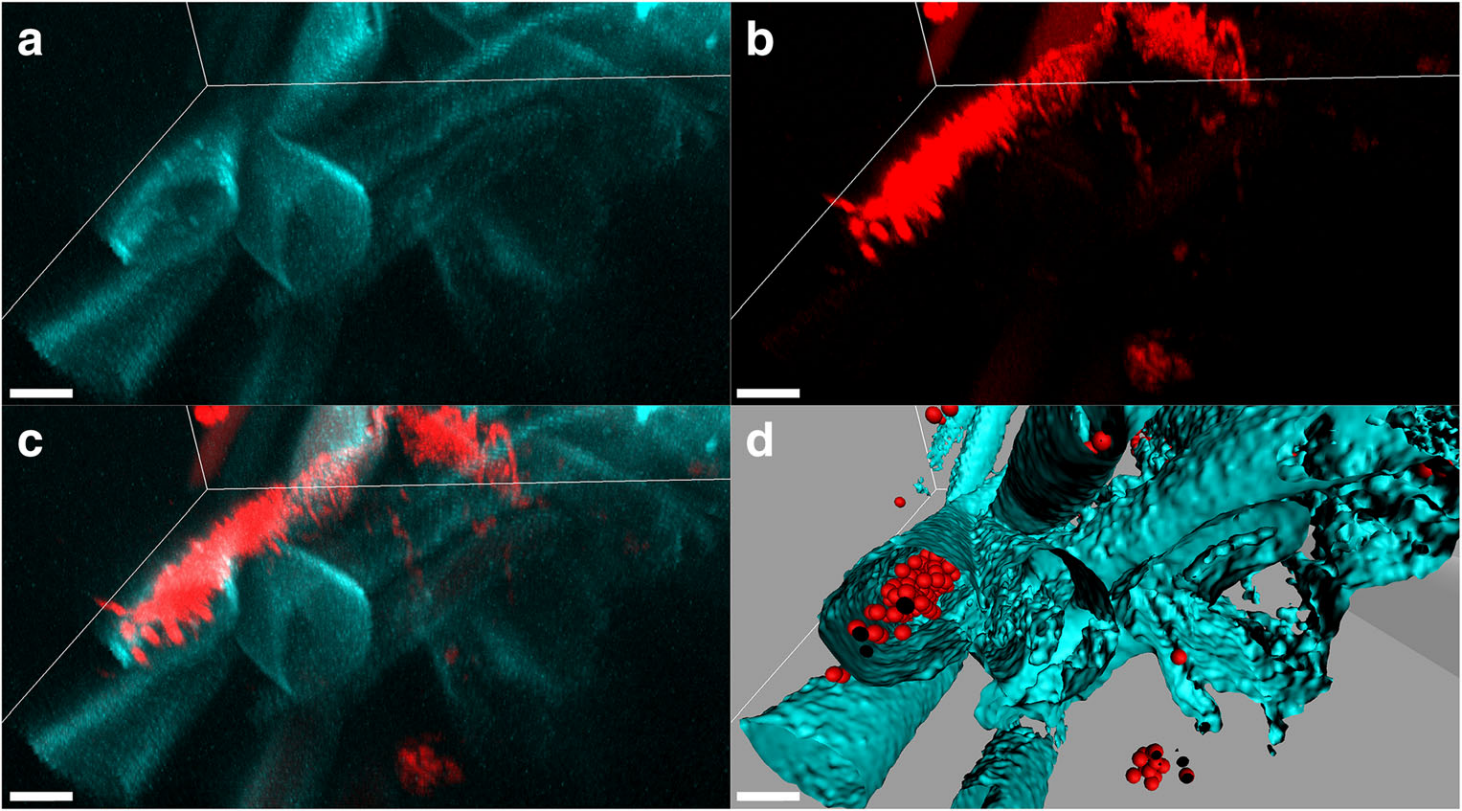
FISH-CLSM images showing the endophytic colonization of banana root hairs by *Pseudomonas palleroniana* Ps006 30 days after inoculum. **a** Root tissue autofluorescence (cyan); **b** signal of the Cy3-labeled EUB338MIX universal probe for bacteria (red); **c** overlay of **a**–**b**; **d** three-dimensional model of **c** showing bacteria as spheres and root tissue as isosurfaces. Scale bars: 10 μm

**Fig. 5:**
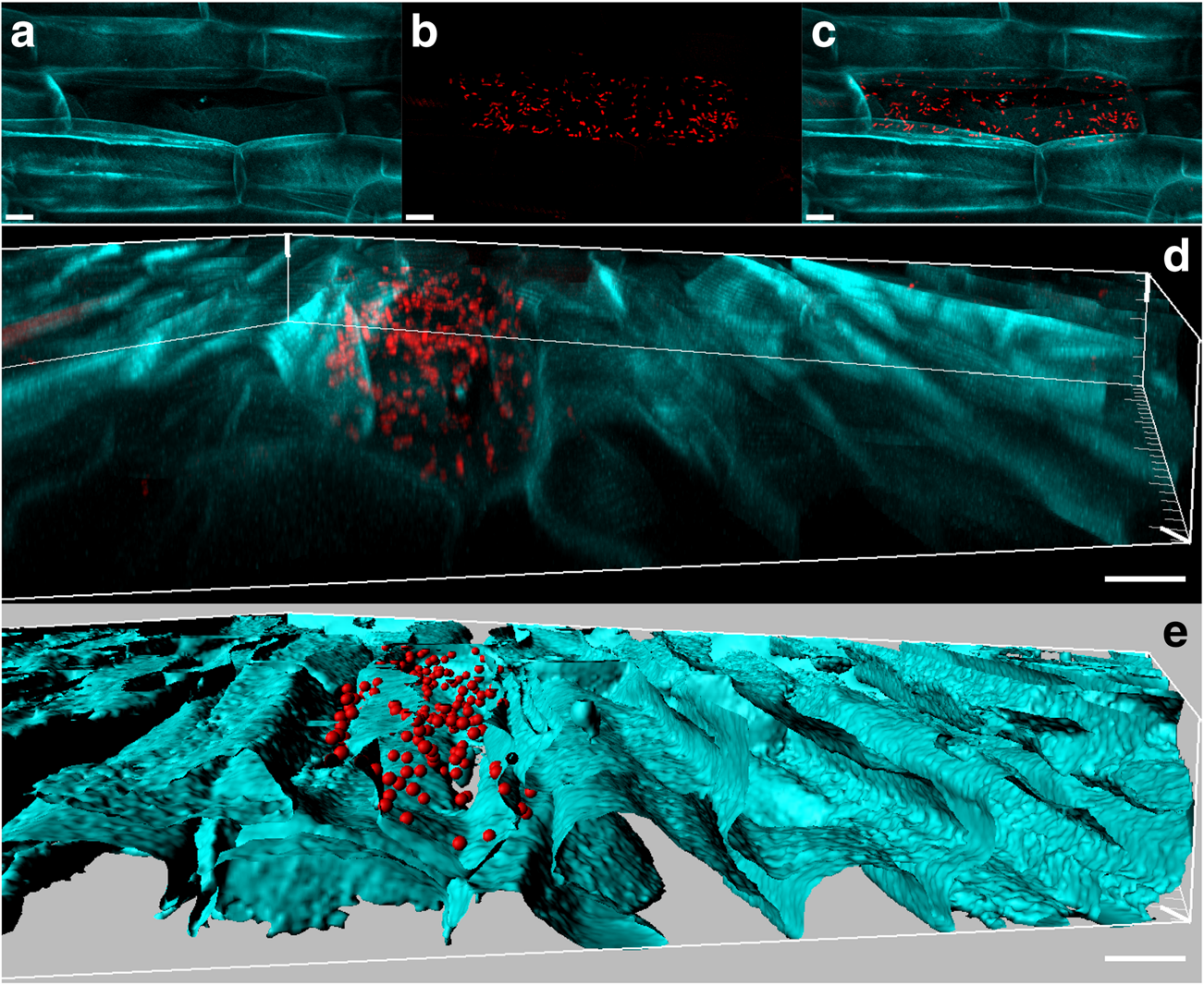
FISH-CLSM images showing the endophytic colonization of banana roots by *Pseudomonas palleroniana* Ps006 96 h after inoculum. **a** Root tissue autofluorescence (cyan); **b** signal of the Cy3-labeled EUB338MIX universal probe for bacteria (red); **c** overlay of **a**–**b**; **d** volume rendering of **c**; **e** three-dimensional model of **d**, showing bacteria as spheres and root tissue as isosurfaces. Scale bars: 10 μm

*Bacillus amyloliquefaciens* Bs006 was very abundant (order of magnitude: 10^10^ cells/cm^3^ plant root tissue) on the rhizoplane (epiphytically) and between root hairs, from 1 h after inoculation (AI) to 48 h AI (Figs. 1a, d and 2). From 96 h AI to 30 d AI, the density strongly decreased to an order of magnitude of 10^7^ cells/cm^3^ plant root tissue (Figs. 1g, j, m and 2). *Pseudomonas palleroniana* Ps006 was detected mostly as single cells (often showing a dividing morphology) until 48 h AI (Figs. 1b, e and 2), markedly less abundant than *B. amyloliquefaciens* Bs006, and localized on the root hairs (order of magnitude: 10^7^ cells/cm^3^ plant root tissue). At 96 h AI and 15 d AI, the density progressively increased (Figs. 1h, k and 2), and it was maximal 30 d AI (order of magnitude: 10^9^ cells/cm^3^ plant root tissue) (Figs. 1n and 2). *B. amyloliquefaciens* Bs006 remained limited to the epiphytic space, colonizing only the root and especially root hair surfaces (Fig. 3). On the other hand, *P. palleroniana* Ps006 clearly colonized banana roots endophytically, starting from 96 h AI. The preferential sites of endophytic colonization were the root hairs (Fig. 4; Fig. S2); however, interesting intracellular colonization of single plant cells within the main root was also observed (Fig. 5). The autofluorescence of those plant cells was evidently lower than the surrounding cells (Fig. 5a), suggesting that they were dead and then became occupied by strain Ps006 bacteria (Fig. 5b–e).

No signal was detected in the non-inoculated roots (Fig. 1c, f, i, l, o) or in the inoculated roots hybridized with the negative FISH–probe NONEUB (Fig. S3).

The trends of the cell densities in the two strains (Fig. 2) were negatively correlated (Spearman’s rho = −0.74, *p* = 0.003), indicating complementarity of their colonization dynamics at a temporal scale.

### In Silico Analysis of the Genome for Plant Growth-Promoting Traits

The genome assembly of *B. amyloliquefaciens* Bs006 has been deposited at GenBank with accession number LJAU00000000 [37]. Details of the genome analysis are reported in Tables S1 and S2. Annotation by RAST (Table S4) revealed 653 factors involved in plant growth-promoting activity and plant–microbe interactions (Table 1). Regarding *P. palleroniana* Ps006, only the factor category “dormancy and sporulation” was more highly represented, due to the ability of *Bacillus* to sporulate (Tables 1 and 2).

**Table 1:** Number of genes involved in plant growth-promoting activity and plant–microbe interactions in *Bacillus amyloliquefaciens* Bs006 and *Pseudomonas palleroniana* Ps006, as detected by in silico genome analysis (RAST, PIFAR, and antiSMASH tools)

Analysis tool	Factor type	Number of factors/genes detected*	
		*Bacillus amyloliquefaciens* Bs006	*Pseudomonas palleroniana* Ps006
RAST 2.0	Dormancy and sporulation	**116**	3
	Cofactors, vitamins, prosthetic groups	**200**	**279**
	Environmental stress response	108	**181**
	Virulence, disease, and defense	67	**149**
	Sulfur metabolism	39	**94**
	Iron acquisition and metabolism	32	**92**
	Phosphorus metabolism	31	**78**
	Pigments	20	**69**
	Nitrogen metabolism	31	**52**
	Potassium metabolism	9	**25**
PIFAR	Antibiotics	**65**	22
	EPS	**18**	15
	MAMP	**16**	4
	Biofilm	**6**	1
	Volatiles	**4**	2
	Hormone	**3**	2
	PCWDE	**4**	1
	Pigment	**4**	0
	Toxin	**3**	0
	MDRs	14	**44**
	Detoxification	4	**25**
	Siderophore	6	**18**
	Adhesion	1	**17**
	Metabolism	10	**12**
	Protease	1	**2**
	Type III effector	0	**1**
	LPS	1	1
antiSMASH	Secondary metabolite biosynthetic cluster	**13**	9

*Numbers in bold indicate the strain with higher abundance. RAST data obtained from [37, 38]. *MDR* microbial drug resistance, *EPS* exopolysaccharides, *MAMP* microbe-associated molecular pattern, *PCWDE* plant cell wall-degrading enzymes

**Table 2:** Genes involved in plant growth-promoting activity and plant–microbe interactions in *Bacillus amyloliquefaciens* Bs006 and *Pseudomonas palleroniana* Ps006, as detected by in silico genome analysis (RAST, PIFAR, and antiSMASH tools)

Analysis tool	Factor type	Factors or genes (number detected)	
		*Bacillus amyloliquefaciens* Bs006	*Pseudomonas palleroniana* Ps006
RAST 2.0	Dormancy and sporulation	Dormancy and sporulation - no subcategory (110), Spore DNA protection (6)	Dormancy and sporulation - no subcategory (3)
	Cofactors, vitamins, prosthetic groups	Folate and pterines (65), Biotin (43), Cofactors, vitamins, prosthetic groups - no subcategory (20), Quinone cofactors (19), Riboflavin, FMN, FAD (18), Coenzyme A (17), Pyridoxine (14), NAD and NADP, Lipoic acid (4)	Folate and pterines (87), Biotin (63), Riboflavin, FMN, FAD (33), NAD and NADP (24), Quinone cofactors (23), Pyridoxine (18), Cofactors, vitamins, prosthetic Groups - no subcategory (14), Coenzyme A (13), Lipoic acid (4)
	Stress response	Oxidative stress (47), Stress response - no subcategory (27), Heat shock (17), Osmotic stress (13), Cold shock (3), Periplasmic stress (1)	Oxidative stress (87), Osmotic stress (35), Stress response - no subcategory (30), Heat shock (16), Cold shock (7), Periplasmic stress (6)
	Virulence, disease, and defense	Resistance to antibiotics and toxic compounds (37), Bacteriocins and RSAP (16), Intracellular invasion and resistance (14)	Resistance to antibiotics and toxic compounds (123), Bacteriocins and RSAP (14), Intracellular invasion and resistance (12)
	Sulfur metabolism	Organic sulfur assimilation (21), Inorganic sulfur assimilation (9), Sulfur metabolism - no subcategory (9)	Organic sulfur assimilation (57), Inorganic sulfur assimilation (27), Sulfur metabolism - no subcategory (10)
	Iron acquisition and metabolism	Iron acquisition and metabolism - no subcategory (17), Siderophores (15)	Iron acquisition and metabolism - no subcategory (55), Siderophores (37)
	Phosphorus metabolism	Phosphorus metabolism - no subcategory (31)	Phosphorus metabolism - no subcategory (78)
	Pigments	Tetrapyrroles (20)	Tetrapyrroles (69)
	Nitrogen metabolism	Nitrogen metabolism - no subcategory (25), Denitrification (6)	Nitrogen metabolism - no subcategory (52)
	Potassium metabolism	Potassium metabolism - no subcategory (9)	Potassium metabolism - no subcategory (25)
PIFAR	Antibiotics	Difficidin (15), Bacillaene (14), Macrolactin (9), Bacilysin (5), Fengycin (5), Lichenysin (4), Subtilin (4), Surfactin (4), Ciscosin (2), Amphisin (1), Bacilysocin (1), Fusaricidin (1)	Arthrofactin (3), Entolysin (3), Massetolide_a_d (3), Orfamide (3), Putisolvin_i_ii (3), Gluconic acid (2), Viscosin (2), Amphisin (1), Fusaricidin (1), Pudacin (1)
	EPS	EPS Bacillus (15), *galU* (1), *gpsX* (1), levan (1)	Alginate (12), *galU* (1), *gpsX* (1), Levan (1)
	MAMP	Teichuronic acid (9), cheA (1), *cheB* (1), *cheC* (1), *cheW* (1), YvyG (1)	*cheA* (1), *cheB* (1), *cheW* (1), *cheY* (1)
	Biofilm	*yhxB* (1), *yqeK* (1), *sipW* (1), *tasA* (1), *ymcA* (1), *yqxM* (1)	*yhxB* (1)
	Volatiles	Acetoin (3), 2,3-Butanediol (1), *budB* (1)	2,3-Butanediol (1), *budB* (1)
	Hormone	Indole-3-acetic acid (3)	Indole-3-acetic acid_2 (2)
	PCWDE	PEC lyase C (2), Cellulase (1), *lipA* (1)	*lipA* (1)
	Pigment	Rubrifacine (4)	–
	Toxin	Syringopeptin (3)	–
	MDRs	Multi Drug Res (6), MatE (5), ACR tran (2)	OEP (22), ACR tran (15), MatE (4), Multidrug resistance (3)
	Detoxification	*cueAR* (1), dps (1), *katB* (1), *katE* (1)	Polymyxin resistance (8), sapABCDF (5), Copper resistance ABCDRS (4), Streptomycin resistance (2), cbb (1), *cueAR* (1), dps (1), *katB* (1), *katE* (1), pip (1)
	Siderophore	Bacillibactin (5), Arthrobactin (1)	Pyoverdine (12), Pyochelin (5), Arthrobactin (1)
	Adhesion	*xadM* (1)	*attC* (1), *attG* (1), Fimbrial (4), Haemagg_act (3), Pilin (1), Usher (6), *xadM* (1),
	Metabolism	*gltBD* (2), *trpCG* (2), *acnB* (1), *aroC* (1), *aroK* (1), *asnB* (1), Citrate_transporter (1), *purD* (1)	*gltBD* (2), *trpCG* (2), acnB (1), *aroC* (1), *aroK* (1), *aroQ* (1), *asnB* (1), Citrate transporter (1), *mqo* (1), *purD* (1)
	Protease	*htrA* (1)	*htrA* (1), Serralysin protease A (1)
	Type III effector	/	*hopB1* (1)
	LPS	*wzt* (1)	*wzt* (1)
antiSMASH	Secondary metabolite biosynthetic cluster	Fengycin (3), Surfactin (3), Bacillaene (1), Bacillibactin (1), Bacilysin (1), Difficidin (1), Macrolactin H (1), Plipastatin (1), Rhizocticin (1)	Pyovedrin (2), Bananamide 1/2/3 (1), Safracin A/B (1), APE Vf (1), Fengycin (1), Lipopolysaccharide (1), Sessilin A (1), Viscosin (1)

*MDR* microbial drug resistance, *EPS* exopolysaccharides, *MAMP* microbe-associated molecular pattern, *PCWDE* plant cell wall-degrading enzymes, *RSAP* ribosomally synthesized antimicrobial peptides

The genome assembly of *P. palleroniana* Ps006 was deposited in GenBank, with accession number LRMR00000000 [38]. Details of the genome analysis are reported in Tables S1 and S3. Annotation by RAST (Table S5) showed 1022 factors or genes involved in plant growth-promoting activity and plant–microbe interactions. All factor categories except dormancy and sporulation were more highly represented in this strain. In particular, the categories “virulence, disease, and defense,” “pigments,” and the category regarding the metabolism of elements generally showed the highest difference (>twofold) (Table 1). Concerning the category “virulence, disease, and defense,” the breakdown of specific factors showed that this difference was due to the highest number of “resistance to antibiotics and toxic compounds” factors (Table 2). On the other hand, bacteriocins and ribosomally synthesized antimicrobial peptides were slightly higher in *B. amyloliquefaciens* Bs006 (Table 2).

Similar to the RAST results, PIFAR identification of genes responsible for functions involved in plant–microbe interactions (Tab. S6-S7) showed that the two rhizobacteria have clearly different functional profiles (Tables 1 and 2). Genes related to drug resistance, detoxification, biosynthesis of siderophores, pigments and vitamins, and metabolism of elements, as well as adhesion potential, were more abundant in *P. palleroniana* Ps006. Meanwhile, *B. amyloliquefaciens* Bs006 showed a notably higher number of genes involved in antibiotic synthesis, exopolysaccharides production, microbe-associated molecular patterns, and biofilm formation (Table 1). In total, consistent with the RAST analysis, a higher number of plant–microbe interaction factors were found in *P*. *palleroniana* Ps006 than in *B. amyloliquefaciens* Bs006, although the difference (167 vs. 160, respectively) was notably less marked. Beyond the differences in abundance, the types of factors or genes detected were clearly different between the two strains (Table 2). Regarding the proportion of individual factors identified in the two bacterial strains, the only incongruity found between the RAST and PIFAR analysis was related to pigments (Tables 1 and 2).

The antiSMASH analysis output was congruent with both previous analysis tools. More biosynthetic clusters of secondary metabolites (basically antimicrobial factors) were identified for *B. amyloliquefaciens* than for *P. palleroniana* (Tables 1 and 2).

## Discussion

This study was designed and built on two previous studies, where we found that the two PGPR strains *B. amyloliquefaciens* Bs006 and *P. palleroniana* Ps006, selected as best promoters of banana cv. Williams [32], induced changes in the host transcripts at different times after inoculation [33]. Therefore, we performed a FISH-CLSM analysis of the same root samples used for transcriptomics and compared the genetic features that were possibly responsible for the differences found.

### Differential Colonization Patterns at Spatial and Temporal Scales

Our investigation confirmed that the two rhizobacteria have different colonization dynamics in banana roots: Bs006 appears very abundant immediately after inoculation until 48 h, and then sharply decreases. Ps006, on the other hand, is less abundant until 48 h, whereupon it increases dramatically. This confirmed our first hypothesis about a temporal scale separation of the two PGPR on banana roots. Moreover, the colonization behavior is also very different: Bs006 colonizes the root epiphytically, while Ps006 becomes clearly endophytic. This confirmed our second hypothesis about the spatial scale separation of the two PGPR on banana roots.

Efficient colonization of plant roots by PGPR is essential to achieving a long-term, beneficial association, for three reasons: (i) if the rhizobacteria do not bind to the root of the plant, the substances that they excrete will spread in the rhizosphere and will be consumed by other soil microorganisms before reaching the plant; (ii) without a firm bond, water can eliminate bacteria from the rhizoplane; and (iii) the root areas that are not associated with PGPR are more vulnerable to phytopathogen colonization [2]. According to our results, *P. palleroniana* Ps006 is more suitable for long-term colonization of banana roots. However, the relatively short occurrence of *B. amyloliquefaciens* Bs006 also clearly affects the banana plants [32, 33]. The observation made at T0 in the current study confirmed our previous observations [32].

*Bacillus amyloliquefaciens* in banana plants stimulated the formation of organic compounds such as fumaric acid that participated significantly in biofilm formation, growth promotion activity, and gene expression [47]. In contrast, *P. palleroniana* Ps006 is endophytic. Consistent with our observations, previous works also detected *P. palleroniana* in the endosphere of banana plants [48, 49]. It seems to be a well-adapted species to the banana endosphere habitat. Additionally, we observed an interesting colonization pattern: the first signs of endophytic colonization by strain Ps006 were the invasion of apparently dead plant cells (Fig. 5). This was observed already at T2, 96 h after inoculation. This might be the invasion strategy used by *P. palleroniana* Ps006 to enter the root endosphere, followed by later colonization of other internal tissues (root hairs). In fact, previous works have shown that root hairs can be colonized endophytically by Gammaproteobacteria that move internally from the main root into the emerging root hairs [50]. The endophytic behavior of *P. palleroniana*, together with its beneficial properties demonstrated in our previous works [32, 33] and in other works on banana [48, 49] and other crops [51, 52], make it an optimal candidate as an efficient and environmentally friendly biofertilizer.

### Differences in the Genetic Background Explain Different Colonization Patterns

**Fig. 6:**
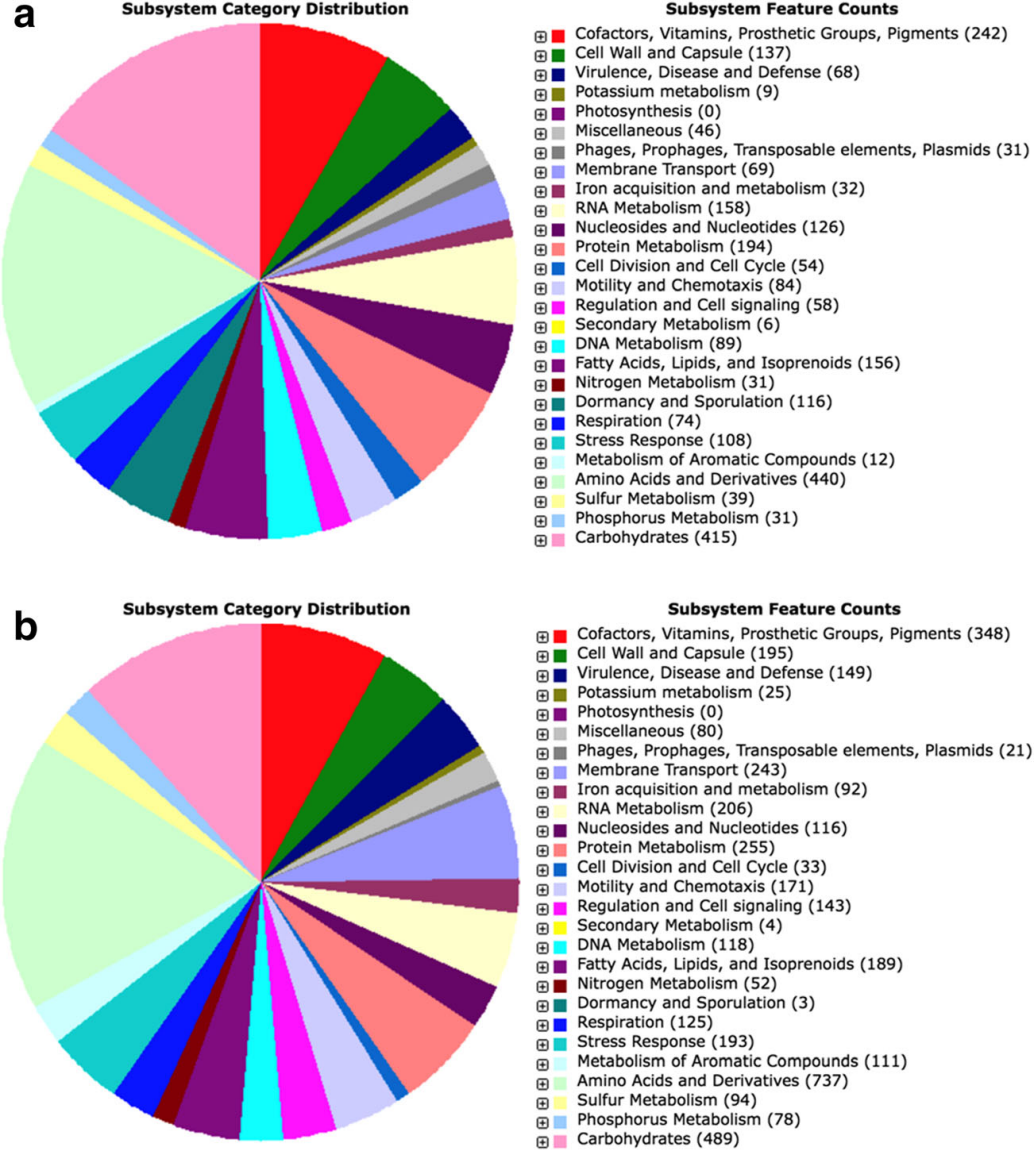
Genes categorized in subsystems or general functions in **a** *Bacillus amyloliquefaciens* Bs006 and **b** *Pseudomonas palleroniana* Ps006, according to the annotation system in RAST 2.0

Despite a generally similar pattern of functional and structural genes in the two genomes (Fig. 6), *P. palleroniana* possesses a higher number of genes/factors involved in plant–microbe interactions compared to *B. amyloliquefaciens*. Moreover, there are notable differences between the two strains regarding the specific genes involved in the potential beneficial effects on the banana host plant. In *P. palleroniana* Ps006, more genes involved in potassium, nitrogen, iron, sulfur, and phosphorus metabolism were found, whereas in *B. amyloliquefaciens* BS006, only sporulation factors were notably more abundant (Tables 1 and 2). As for plant stress and defense, PGPR can aid in active plant growth in conditions of abiotic or biotic stresses [53]. PGPR produce repressive substances that increase the natural resistance to phytopathogens and pests [9], such as hydrolytic enzymes (chitinases, cellulases, and proteases), as well as various antibiotics, volatile organic compounds (VOCs), exopolysaccharides (EPS), and siderophores that protect plants against phytopathogens [9, 54]. The *P. palleroniana* Ps006 strain used in the current study has a genome that contains more genes for resistance to antibiotics and toxic compounds (Tables 1 and 2). Further, the in silico genome analysis and the online tool PIFAR were used to detect the presence of genes involved in plant–microbe interactions; the results were mostly coherent with those of RAST (except for pigments). Similar to the RAST analysis, PIFAR also highlighted notable differences between the two strains (Tables 1 and 2). Considering the antiSMASH analysis, we confirm that the three analysis tools generated coherent outputs among one another, with very few incongruities.

All these distinctive genetic traits explain the different colonization behaviors observed between *P. palleroniana* Ps006 and *B. amyloliquefaciens* Bs006 on banana roots and substantiate the various effects on plant growth and gene expression found in our previous works [33, 34]. In particular, the endophytic behavior of *P. palleroniana* Ps006 seems to be associated with a higher number of genes involved in drug resistance, detoxification, adhesion, siderophore/pigment/vitamin synthesis and, in general, nutrient metabolism; on the other hand, antibiotic synthesis, exopolysaccharides production, microbe-associated molecular patterns, and biofilm formation potential seem to be more related to *B. amyloliquefaciens* and banana root surface (although this association was only short-term in our study).

## Conclusions

Studies like this are necessary to understand the ecology and the interactions of PGPR with the inoculated plants at spatial and temporal scales. The obtained information can then be used to optimize the protocol of biofertilizer formulation and application to maximize its efficacy. In this work, we showed a complementarity colonization dynamic between *B. amyloliquefaciens* Bs006 and *P. palleroniana* Ps006 (earlier vs. later), as well as complementarity in habitat preference (epiphytic vs. endophytic). The different genetic background related to functions involved in plant–microbe interactions supports and substantiates the different colonization dynamics observed. Our results suggest that the two strains could theoretically be inoculated on the plant simultaneously, without competing for the niche. This means that they might be used together in a single biofertilizer formulation. Moreover, this appealing hypothesis needs to be tested in the future with co-inoculation experiments, which might also reveal further beneficial effects on banana growth by synergistic microbe–microbe interactions.

## Electronic supplementary material

ESM 1 (PDF 3125 kb)

ESM 2 (XLSX 2374 kb)

## Data Availability

The genome sequences of *Bacillus amyloliquefaciens* Bs006 and *Pseudomonas palleroniana* Ps006 analyzed during the current study are available in the GenBank repository [www.ncbi.nlm.nih.gov/nuccore/LJAU00000000;www.ncbi.nlm.nih.gov/nuccore/LRMR00000000].
