# Supplementary material for: Complementary Dynamics of Banana Root Colonization by the Plant Growth-Promoting Rhizobacteria Bacillus amyloliquefaciens Bs006 and Pseudomonas palleroniana Ps006 at Spatial and Temporal Scales
Source: Microb Ecol. 2020 Aug 11;80(3):656–68. doi: 10.1007/s00248-020-01571-0 (PMC7476998; doi:10.1007/s00248-020-01571-0)
Supplement: Supplementary file 1 — (PDF 3125 kb) [file 248_2020_1571_MOESM1_ESM.pdf]

Supplementary material to:

Complementary dynamics of banana root colonization by the plant growth-promoting rhizobacteria *Bacillus amyloliquefaciens* Bs006 and *Pseudomonas palleroniana* Ps006 at spatial and temporal scales

Gamez Rocío M., Ramirez Sandra, Montes Martha, Cardinale Massimiliano*

* massimiliano.cardinale@unisalento.it

- **Fig. S1: Micropropagation procedure of *Musa acuminata* plants using the apical meristem**
- **Fig. S2: Fluorescence microscopy images showing the endophytic colonization of banana root hairs by *Pseudomonas palleroniana* Ps006, 30 days after inoculum**
- **Fig. S3: FISH negative control**
- **Tab. S1: Details of the genomes of *Bacillus amyloliquefaciens* Bs006 and *Pseudomonas palleroniana* Ps006**
- **Tab. S2: Categories of orthologous genes (COG) in the genome of *Bacillus amyloliquefaciens* Bs006 - (included as excel file)**
- **Tab. S3: Categories of orthologous genes (COG) in the genome of *Pseudomonas palleroniana* Ps006 - (included as excel file)**
- **Tab. S4: Full table of RAST 2.0 analysis of *Bacillus amyloliquefaciens* Bs006 - (included as excel file)**
- **Tab. S5: Full table of RAST 2.0 analysis of *Pseudomonas palleroniana* Ps006 - (included as excel file)**
- **Tab. S6: Full table of PIFAR analysis of *Bacillus amyloliquefaciens* Bs006 - (included as excel file)**
- **Tab. S5: Full table of PIFAR analysis of *Pseudomonas palleroniana* Ps006 - (included as excel file)**

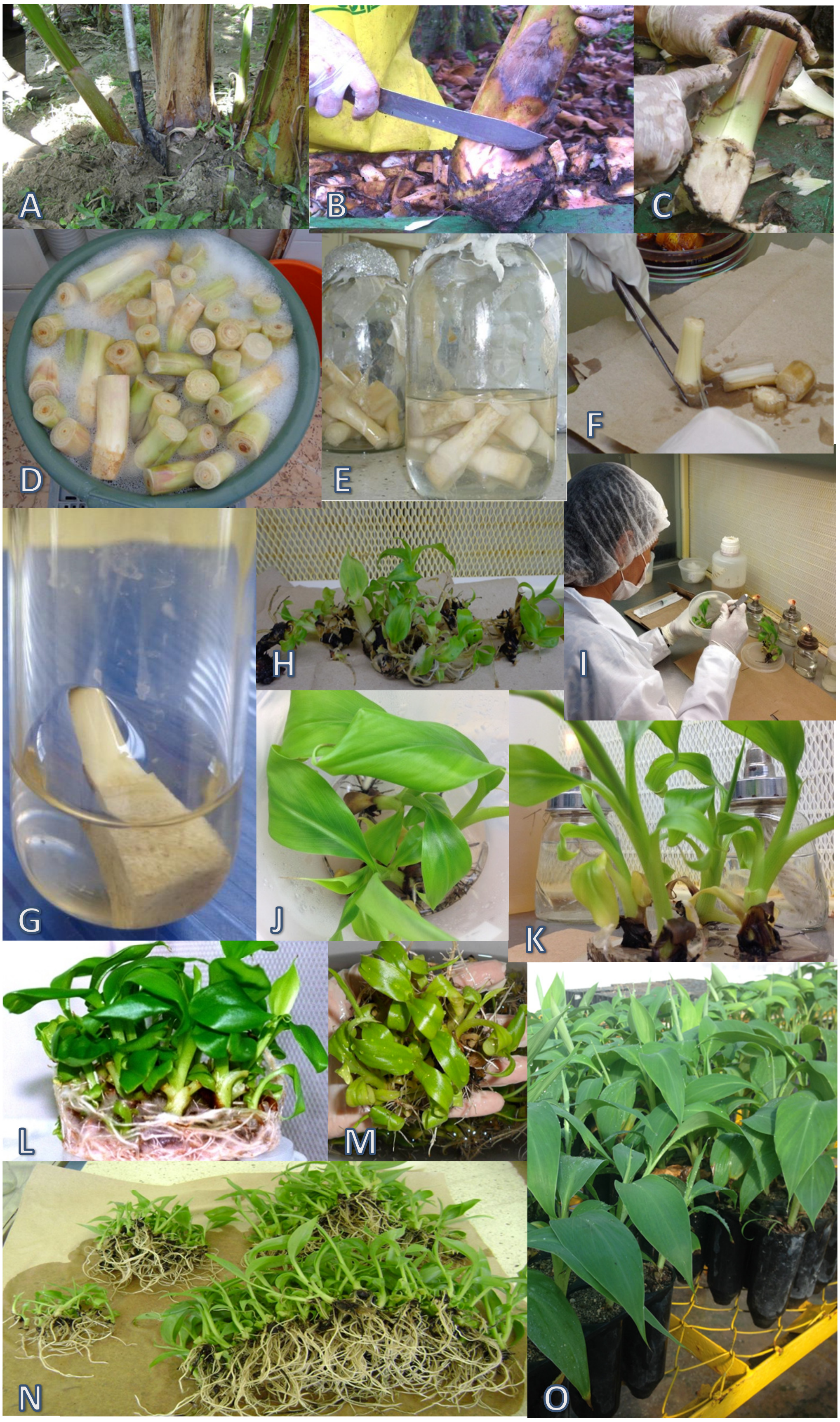
A
B
C
D
E
F
G
H
I
J
K
L
M
N
O

Figure S1: Micropropagation procedure of *Musa acuminata* plants using the apical meristem. A: Extraction of corms in the field, B: Elimination of roots. C: Reduction of layers of the pseudostem, D: Successive washing and disinfection in detergent (iodine and chlorine). E: Continuation of reductions and disinfections in corm cores of approximately 10 cm. F: Final reduction of the apical meristem, G: Establishment in modified Murashige and Skoog (MS) culture medium. H: Individualization of the generated plants. I: Explant multiplication phase. J-K: Seedlings generated in the multiplication phase. L: *In vitro* rooting. In this phase, the rhizobacteria were applied for confocal microscopy analysis. M: Plant washing and elimination of surplus culture medium. N: Size classification. O: Sowing and hardening under *ex vitro* conditions to acclimatize the plants.

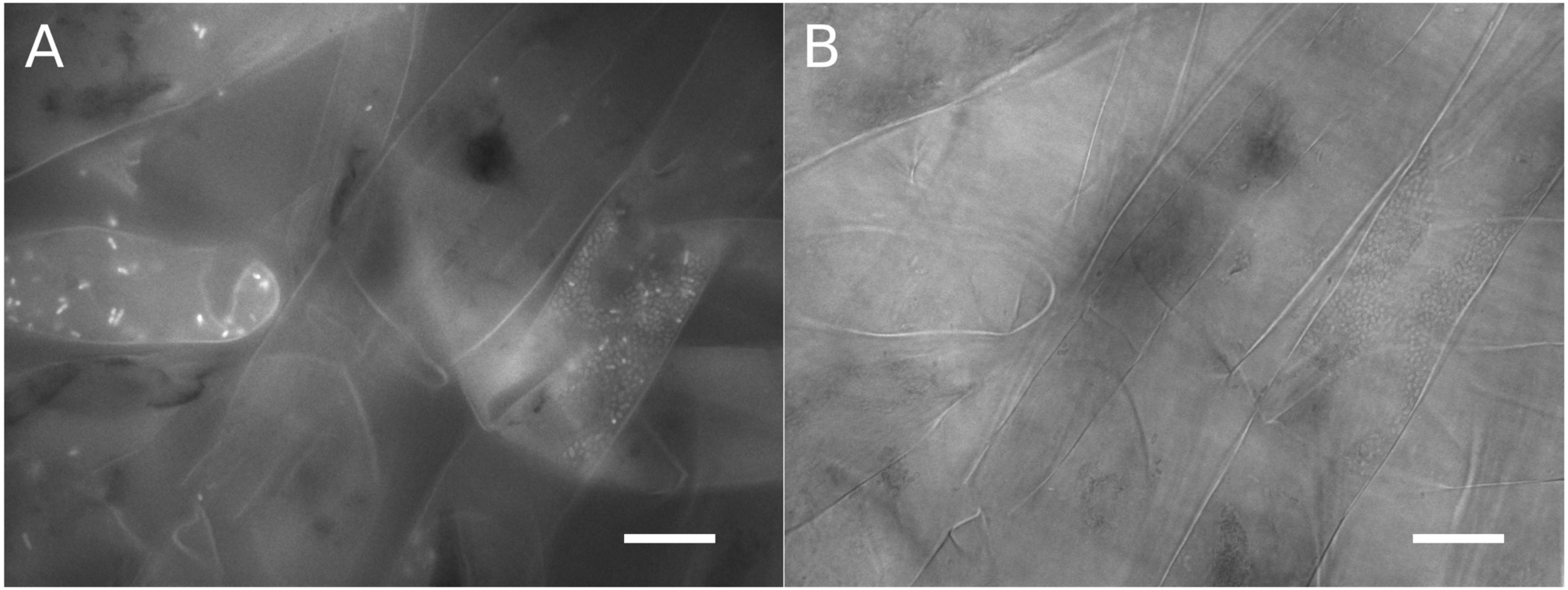

Figure S2: **Fluorescence microscopy images showing the endophytic colonization of banana root hairs by *Pseudomonas palleroniana* Ps006, 30 days after inoculum.** A) Fluorescent image (Zeiss Axioplan microscope; Filter Set 15); B) Correspondent bright field image. Samples were stained by FISH using the EUB338MIX probe labelled with Cy3. Scale bars: 15 µm.

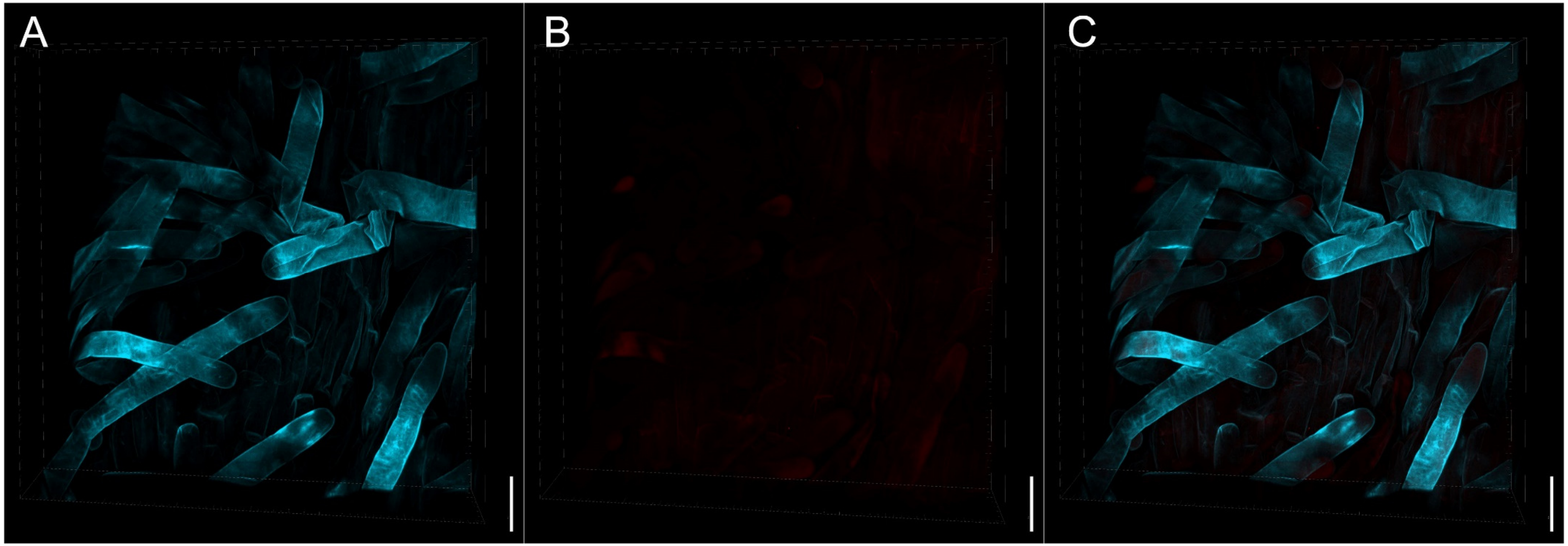

Figure S3: **FISH negative control**. FISH-CLSM images showing inoculated banana roots stained by FISH, by using the non-sense probe NONEUB. Only root tissue autofluorescence (A), and no probe-conferred signal (B), was detected. C) Overlap of A and B. Scale bars: 30 μm.

Table S1 Genome details of *Bacillus amyloliquefaciens* Bs006 (data from [37]) and *Pseudomonas palleroniana* Ps006 (data from [38])

Strain	bp length	GC content	N. of contigs	cds	N. of rRNA	N. of tRNA	N. of ncRNA
Bacillus amyloliquefaciens Bs006	4,173,094	46.4%	86	3,998	6	67	1
Pseudomonas palleroniana Ps006	6,250,362	60.3%	47	5,474	4	61	4
